# Supplementary material for: Prediction of Cognitive Decline in Parkinson’s Disease Using Clinical and DAT SPECT Imaging Features, and Hybrid Machine Learning Systems
Source: Diagnostics (Basel). 2023 May 10;13(10):1691. doi: 10.3390/diagnostics13101691 (PMC10217464; doi:10.3390/diagnostics13101691)
Supplement: Supplementary file 1 [file diagnostics-13-01691-s001.zip › diagnostics-2303892-supplementary.pdf]

SUPPLEMENTAL MATERIAL

A. DAT SPECT image characteristics:

Imaging was performed at screening 4.0 ± 0.5 h after injection of DAT SPECT (123I-Ioflupane; 111-185 MBq) according to the imaging technical operations manual (www.ppmi-info.org). Thyroid update was prevented by pre-treating subjects with either a saturated iodine solution (10 drops in water) or perchlorate (1000 mg) before receiving an injection. The data set included 128 × 128 raw SPECT projection data acquired every 3 degrees, 120 projections, 20% symmetric photopeak windows centered on 159 keV and 122 keV, and a total scan duration of 30-45 minutes. A central core imaging laboratory developed a program for a technical qualification, quality assurance, and ongoing camera quality control to ensure technical standardization across multiple sites and cameras used. The same protocol used for PPMI subjects was used to fill an anthropomorphic striatal phantom with 123-I. This phantom was used to test the accuracy and resolution of the reconstructed image volume, as well as to create a site-specific attenuation correction factor (mu) for use with the data at the imaging core lab.

The raw projection data was transferred from the sites to the central core imaging laboratory for quality control, which included motion assessment, standardized reconstruction, attenuation correction, and quantification. The raw projection data from a SPECT scan were imported into a HERMES (Hermes Medical Solutions, Skeppsbron 44, 111 30 Stockholm, Sweden) system for iterative (HOSEM) reconstruction. In other words, this was done for all imaging data to ensure that the reconstructions were consistent.

Iterative reconstruction was performed with no filtering. The reconstructed HOSEM files were then sent to PMOD (PMOD Technologies, Zurich, Switzerland) for further processing. Attenuation correction ellipses were drawn on the images, and a Chang 0 attenuation correction was applied to images using a site-specific mu derived empirically from phantom data collected during trial site initiation. After that, standard 3D Gaussian post-smoothing (6.0 mm FWHM) was used. Furthermore, to ensure consistent anatomical alignment across scans, these files were normalized to a SPECT ioflupane reference template in standard Montreal Neurologic Institute (MNI) space. The most robust normalizations for the ioflupane DAT-SPECT image volumes were obtained using intramodality spatial normalization using the standard template. The transaxial slice with the highest striatal uptake was then identified, and the eighth hottest striatal slices around it were averaged to produce a single-slice image. Finally, the images collected from the PPMI

database share characteristics such as image dimensions (X, Y, Z): 91 × 109 × 91, voxel dimensions (X, Y, Z): 2 × 2 × 2 Millimeters, etc.

B. Machine learning methods

1. Feature selection algorithms

In machine learning, pattern recognition, and image processing, Feature Extraction starts from an initial set of measured data and builds derived values (features) intended to be informative and non-redundant, facilitating the subsequent learning and generalization steps, and in some cases leading to better human interpretations. Feature extraction is related to dimensionality reduction (1). In this study, analysis of variance (ANOVA) as a statistical algorithm is used to select the most relevant features. This technique allows us to specify the impact of a single feature on outcomes depending on many factors as well as it ranks the features based on priorities. (2)

2. Classifier algorithms (CAs)

Below, we elaborate on the **8** classifier machines used in our present work.

2.1. AdaBoost classifier (Adaboost)

AdaBoost, short for Adaptive Boosting, is an ensemble method that starts by fitting a base estimator on the data and then fits additional copies of the base estimator on the same data with the weights of data samples adjusted according to the error of the current prediction. In this way, subsequent estimators focus more on the cases where the error is larger (3).

2.2. Bagging classifier (BagC)

Bagging is based on bootstrapping and aggregating concepts, so it incorporates the benefits of both approaches. Bootstrapping is based on random sampling with replacement. Therefore, taking a bootstrap replicate Xb =(Xb1, Xb2, . . ., Xbn) (random selection with replacement) of the training set X =(X1, X2, . . ., Xn), one can sometimes avoid or get less misleading training objects in the bootstrap training set.

Consequently, a classifier constructed on such a training set may have a better performance. Aggregating means combining classifiers. Often a combined classifier gives better results than individual classifiers, because of combining the advantages of the individual classifiers in the final solution. Therefore, bagging might be helpful to build a better classifier on training sample sets with misleaders (4).

2.3 Extra tree classifier (ETC)

An extremely randomized tree classifier, known as Extra-Tree (ET) classifier, ET is an ensemble classification method of decision tree classifier, which differs from classic decision trees in the way they are built. This means that in order to find the best split to separate the samples of a node into two groups, random splits are drawn from each of the randomly selected features, namely max_features, from which the best split will be chosen. As such, the ET classifier fits with various sub-samples of a dataset by averaging out the variance problems of a single decision tree method, which helps improve the predictive accuracy and the control of over-fitting (5).

2.4. Gradient boosting classifier (GBC)

Gradient boosting is another boosting ensemble method that treats boosting as an optimization problem on a differentiable cost function (3).

2.5. Extreme gradient boosting classifier (XGBC)

XGBoost, which stands for Extreme Gradient Boosting, is a scalable, distributed gradient-boosted decision tree (GBDT) machine learning library. It provides parallel tree boosting and is the leading machine learning library for regression, classification, and ranking problems. It's vital to an understanding of XGBoost to first grasp the machine learning concepts and algorithms that XGBoost builds upon: supervised machine learning, decision trees, ensemble learning, and gradient boosting. Supervised machine learning uses algorithms to train a model to find patterns in a dataset with labels and features and then uses the trained model to predict the labels on a new dataset's features. (6)

2.6. K-Nearest neighbor classifier (KNNC)

K Nearest Neighbor (KNN) Algorithm is a classification algorithm It uses a database which is having data points grouped into several classes and the algorithm tries to classify the sample data point given to it as a classification problem. KNN does not assume any underlying data distribution and so it is called non-parametric. The advantages of KNN algorithm are the following: it is a simple technique that is easily implemented. Building the model is cheap. It is an extremely flexible classification scheme and well-suited for Multi-modal classes. Records are with multiple class labels. The error rate is at most twice that of Bayes error rate. It can sometimes be the best method**.** Disadvantages of KNN are the following: classifying unknown records are relatively expensive. It requires distance computation of k-nearest neighbors. With the growth in training set size, the algorithm gets computationally intensive, Noisy/irrelevant features will result in degradation of accuracy (7).

2.7. Multilayer perceptron classifier (MLPC)

The multilayer perceptron consists of a system of simple interconnected neurons or nodes**.** The nodes are connected by weights and output signals which are a function of the sum of the inputs to the node modified by a simple nonlinear transfer, or activation, function. It is the superposition of many simple nonlinear transfer functions that enables the multilayer perceptron to approximate extremely non-linear functions (8).

2.8. Random forests classifier (RFC)

A random forest (RF) is an ensemble classifier and consists of many DTs similar to the way a forest is a collection of many trees. DTs that are grown very deep often cause overfitting of the training data, resulting in a high variation in classification outcome for a small change in the input data. They are very sensitive to their training data, which makes them error-prone to the test dataset. The different DTs of an RF are trained using the different parts of the training dataset. To classify a new sample, the input vector of that sample is required to pass down with each DT of the forest. Each DT then considers a different part of that input vector and gives a classification outcome. The forest then chooses the classification of having the most 'votes' (for discrete classification outcome) or the average of all trees in the forest (for numeric classification outcome). Since the RF algorithm considers the outcomes from many different DTs, it can reduce the variance resulting from the consideration of a single DT for the same dataset (9).

Reference

1. Optimization of data-driven filterbank for automatic speaker verification - ScienceDirect [Internet]. [cited 2022 Nov 14]. Available from: https://www.sciencedirect.com/science/article/abs/pii/S1051200420301408

2. An ANOVA approach for statistical comparisons of brain networks | Scientific Reports [Internet]. [cited 2022 Nov 14]. Available from: https://www.nature.com/articles/s41598-018-23152-5

3. Machine Learning for Predicting the Band Gaps of ABX3 Perovskites from Elemental Properties | The Journal of Physical Chemistry C [Internet]. [cited 2022 Nov 14]. Available from: https://pubs.acs.org/doi/abs/10.1021/acs.jpcc.9b11768

4. Bagging, Boosting and the Random Subspace Method for Linear Classifiers | SpringerLink [Internet]. [cited 2022 Nov 14]. Available from: https://link.springer.com/article/10.1007/s100440200011

5. (PDF) Hybridization of SLIC and Extra Tree for Object Based Image Analysis in Extracting Shoreline from Medium Resolution Satellite Images [Internet]. [cited 2022 Nov 14]. Available from: https://www.researchgate.net/publication/322050216_Hybridization_of_SLIC_and_Extra_Tree_for_Object_Based_Image_Analysis_in_Extracting_Shoreline_from_Medium_Resolution_Satellite_Images

6. What is XGBoost? [Internet]. NVIDIA Data Science Glossary. [cited 2022 Nov 14]. Available from: https://www.nvidia.com/en-us/glossary/data-science/xgboost/

7. A Quick Review of Machine Learning Algorithms | Semantic Scholar [Internet]. [cited 2022 Nov 14]. Available from: https://www.semanticscholar.org/paper/A-Quick-Review-of-Machine-Learning-Algorithms-Ray/8db8166249dfb94dd8d52f88d27917b5755ae049

8. Gardner MW, Dorling SR. Artificial neural networks (the multilayer perceptron)—a review of applications in the atmospheric sciences. Atmos Environ. 1998 Jan 1;32:2627–36.

9. Comparing different supervised machine learning algorithms for disease prediction | BMC Medical Informatics and Decision Making | Full Text [Internet]. [cited 2022 Nov 14]. Available from: https://bmcmedinformdecismak.biomedcentral.com/articles/10.1186/s12911-019-1004-8
